# Generation of the Cdk5 activator p25 is a memory mechanism that is affected in early Alzheimer’s disease

**DOI:** 10.3389/fnmol.2014.00036

**Published:** 2014-05-01

**Authors:** K. Peter Giese

**Affiliations:** Centre for the Cellular Basis of Behaviour, James Black Centre, King’s College LondonLondon, UK

**Keywords:** Alzheimer’s disease, memory, synaptic plasticity, synaptic degeneration, p25, p35, Cdk5

## Abstract

About 15 years ago it was proposed that generation of the truncated protein p25 contributes to toxicity in Alzheimer’s disease (AD). p25 is a calcium-dependent degradation product of p35, the principal activator of cyclin-dependent kinase 5 (Cdk5). The biochemical properties of p25 suggested that its generation would cause Cdk5 overactivation and tau hyperphosphorylation, a prerequisite for neurofibrillary tangle (NFT) formation. Whilst this model was appealing as it explained NFT formation, many laboratories could not confirm the finding of increased p25 generation in brain from AD patients. On the contrary, it emerged that p25 levels are reduced in AD. This reduction occurs primarily in the early stages of the disease. Further, p25 generation in the mouse hippocampus is associated with normal memory formation and p25 overexpression enhances synaptogenesis. Therefore, it transpires that p25 generation is a molecular memory mechanism that is impaired in early AD. I discuss the prospect that investigation of p25-regulated proteins will shed light into mechanisms underlying synaptic degeneration associated with memory decline in AD.

## INTRODUCTION

**FIGURE 1:**
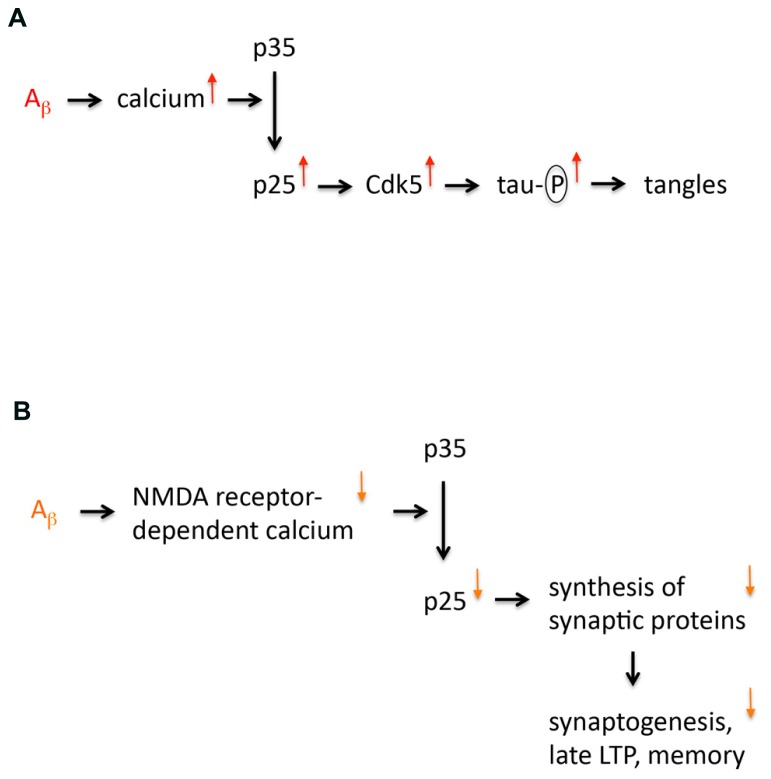
**Models of p25 dysregulation in Alzheimer’s disease. (A)** The first model implicating p25 in AD is shown. This model proposes that p25 expression is increased in AD. This increased expression is caused by amyloid oligomers that increase calcium signaling enhancing cleavage of p35 into p25. Increased p25 expression leads to an overactivation and mislocalization of Cdk5, which results in tau hyperphosphorylation, which is a prerequisite for neurofibrillary tangle formation and neurodegeneration. **(B)** Revised model of p25 dysregulation in AD. This model proposes that p25 expression is not increased but reduced in AD. This decreased expression is caused by amyloid oligomers that decrease NMDA receptor-dependent calcium signaling at the synapse (due to internalization and desensitization of NMDA receptors) impairing cleavage of p35 into p25. Decreased p25 expression reduces synthesis of particular synaptic proteins, which affects synaptogenesis, late LTP and memory formation.

In the late Nineties Li-Huei Tsai’s lab published a paper proposing that the truncated cyclin-dependent kinase 5 (Cdk5) activator p25 is specifically formed in sporadic Alzheimer’s disease (AD; Patrick et al., 1999). This finding evoked a lot of interest because it appeared to provide a missing mechanism for the amyloid cascade hypothesis. It supplied a link between abnormal processing of amyloid precursor protein (APP) and tau hyperphosphorylation, a prerequisite of neurofibrillary tangle (NFT) formation. The model (**Figure 1**) was as follows: Abnormal APP processing generates amyloid peptides that oligomerize thereby enhancing calcium signaling (Kawahara, 2010). This would result in activation of the calcium-dependent protease calpain leading to cleavage of p35 into p25. Both p35 and p25 are activators of Cdk5, a major tau kinase (Angelo et al., 2006). p25 formation would overactivate Cdk5, since p25 is more stable than p35 due to lack of an ubiquitination site (Angelo et al., 2006). Additionally, p25 is cytosolic, in contrast to plasma membrane-bound p35. Therefore, p25 formation can activate Cdk5 in locations that are not close to the membrane. Consequently, p25 formation is well positioned to cause hyperphosphorylation of “free” tau that is not associated with microtubules. However, research over the last 15 years has shown that this model is not correct because p25 levels are not increased in AD. Instead it has emerged that p25 formation is a memory mechanism that is impaired in early AD. Here, I discuss the key evidence leading to this conclusion and pose some outstanding questions.

## p25 EXPRESSION IS NOT INCREASED BUT REDUCED IN AD

Two years after the Patrick et al. (1999) paper another postmortem analysis was published, which did not confirm the claimed upregulation of p25 expression in postmortem AD (Yoo and Lubec, 2001). In fact this study showed a significant *downregulation* of p25 expression in AD forebrain. This decreased p25 expression was not simply due to neuronal loss, as it was adjusted to the expression of a neuronal marker. However, the downregulation of p25 expression was not discussed by the authors, possibly because p25 expression was considered toxic in line with Tsai’s lab. Instead, the key point of discussion was that p25 upregulation was not reproducible in postmortem AD tissue. It was realized that postmortem delay is a variable to consider, since p25 can be formed from degradation of p35 before the tissue is frozen (Kusakawa et al., 2000). Thus, the failure to detect p25 upregulation was attributed to postmortem delay. However, p25 expression was downregulated in the study by Yoo and Lubec (2001) indicating that the postmortem delay was not too long. In fact, the postmortem delay was similar to that previously reported by Patrick et al. (1999). Two further labs also found evidence for a downregulation of p25 expression in AD. In one study there was a trend for downregulation (*p* = 0.06) (Tandon et al., 2003) and in the other study p25 levels as well as p35 levels were reduced by 50% (Taniguchi et al., 2001). Recently, my lab also performed a postmortem expression analysis (Engmann et al., 2011a). In our work we not only compared p25 and p35 expression in severe AD (Braak stages 5–6) as in the previously published postmortem analyses (Patrick et al., 1999; Taniguchi et al., 2001; Yoo and Lubec, 2001; Tseng et al., 2002; Tandon et al., 2003), but we also examined expression in mild AD (Braak stages 1–2). We found that p25 and p35 expression are reduced in mild AD and there is no additional reduction in severe AD. Our work also suggests that the sample preparation used by Tsai’s lab may have biased their result, because they considered only protein that was remaining after a long highspeed centrifugation step which biases p25/p35 (Engmann et al., 2011a). Taken together, various postmortem analyses have revealed that p25 expression is not increased in AD as widely thought; instead p25 expression is actually reduced. Reduced p25 in AD is in agreement with amyloid-induced synaptic toxicity, since amyloid oligomers eventually decrease calcium signaling at the synapse due to internalization and desensitization of N-methyl-D-aspartate (NMDA) receptors (Palop and Mucke, 2010; Paula-Lima et al., 2013).

## MOUSE MODELS OF p25 EXPRESSION

Motivated by the Patrick et al. (1999) paper, various transgenic mouse models were generated expressing high levels of p25, in most cases during embryonic development, which confounds the analysis as Cdk5 has developmental functions (see, Giese et al., 2005). High-level p25 expressing mouse models suffer from neurodegeneration (e.g., Fischer et al., 2005). Thus, it is clear that overexpression of p25 can be neurotoxic. This is likely due to pathological processing of key disease proteins such as tau and APP, which may be an artifact of overexpression. However, this model of neurotoxicity is not relevant to AD, since p25 levels are not increased but reduced in AD.

## p25 GENERATION DURING SPATIAL MEMORY FORMATION

How can p25 expression be reduced in AD? A reduction in p25 expression indicates that p25 formation occurs under normal physiological conditions, and that such physiological p25 generation is impaired in AD. In agreement with this, spatial memory formation is associated with p25 generation in the mouse hippocampus (Engmann et al., 2011a). Importantly, behavioral controls did not show any p25 generation in the mouse hippocampus in the absence of memory formation.

The importance of p25 generation for spatial memory has been tested in various mutant mouse lines. First, mutant mice expressing low levels of p25 under control of the αCaMKII promoter which is active in postnatal forebrain, show improved spatial memory formation (Angelo et al., 2003; Ris et al., 2005). Second, short-lasting overexpression of p25 under inducible control of the αCaMKII promoter also enhances spatial memory formation (Fischer et al., 2005). Third, heterozygous p35 knockout mice, which are thought to generate less p25 than wild-type mice, have impaired spatial reversal learning (Engmann et al., 2011b). Taken together, these studies suggest that p25 generation is important for spatial memory formation. In future, it will be important to confirm this hypothesis with p35 knock-in mutants that lack the calpain cleavage site, precluding p25 generation.

## HOW DOES p25 GENERATION IMPROVE MEMORY FORMATION?

Overexpression of physiological levels of p25, or transient expression of high levels of p25 enhance spatial memory formation (Angelo et al., 2003; Fischer et al., 2005; Ris et al., 2005). In these models p25 overexpression increases synapse density (Fischer et al., 2005; Engmann et al., 2011a) and the late phase of hippocampal CA1 LTP (Ris et al., 2005). These findings suggest that p25 generation leads to increased protein synthesis that is required for late LTP and synaptogenesis. This idea was tested by analysis of the synaptic proteome of p25 mutants in comparison to control littermates (Engmann et al., 2010, 2011a). It was found that p25 overexpression increases the expression of fewer than 20 synaptic proteins out of approximately 1000 proteins analyzed. Thus, p25 generation during spatial memory formation may increase synthesis of a few synaptic proteins that may induce synaptogenesis and contribute to the late phase of LTP. It is conceivable that p25 acts as a signal from synapse to nucleus (see, O’Hare et al., 2005) to stimulate expression of genes encoding synaptic proteins. Alternatively, p25 may induce translation of mRNAs that are located at synapses, although there is currently no evidence that Cdk5 regulates local protein synthesis. In any case, p25 generation seems to be implicated in specific protein synthesis that is required for synaptogenesis and long-lasting LTP.

## THE IMPACT OF p25 DOWNREGULATION IN AD

In AD synapse loss, which precedes neuronal loss, correlates best with impaired memory formation (Arendt, 2009; Serrano-Pozo et al., 2012). Thus, synaptic dysfunction is fundamental for the early stages of AD (see also, Selkoe, 2002). The downregulation of p25 expression in AD is expected to contribute to synaptic dysfunction in early AD, because (i) p25 generation is linked with synaptogenesis and LTP (Fischer et al., 2005; Ris et al., 2005; Engmann et al., 2011a,b), (ii) downregulation of p25 generation occurs in the early stages of AD (Engmann et al., 2011a), and (iii) p25 generation is associated with memory formation. Since p25 generation regulates the expression of particular synaptic proteins (Engmann et al., 2011a), p25-regulated synaptic proteins may be reduced in their expression in early AD and this may lead to synaptic degeneration. This idea was tested in the case of optic atrophy 1 (OPA1) and septin 7, both being p25-regulated proteins (Engmann et al., 2011a). OPA1 is a mitochondrial protein involved in mitochondrial fusion as well as spinogenesis (Cipolat et al., 2004; Wang et al., 2009). Septin 7 is a GTP-binding protein that is localized to the spine neck, where it controls spinogenesis and morphology of the spines (Tada et al., 2007; Xie et al., 2007). Whilst the expression of septin 7 does not appear to be altered in postmortem AD brain, OPA1 expression is reduced in the hippocampus in early AD (Engmann et al., 2011a). This suggests that dysregulation of p25-regulated synaptic proteins contributes to synaptic dysfunction in early AD. In future, the functional impact of these dysregulations will need to be determined.

Cdk5 and glycogen synthase 3 (GSK-3) are thought to be the main tau kinases (Engmann and Giese, 2009). In AD reduction of p25 and p35 expression in hippocampus correlates with tau hyperphosphorylation (Engmann et al., 2011a). Reduced p25 and p35 expression should result in decreased Cdk5 activity in AD and consequently Cdk5 may not be the critical tau kinase. Instead, reduced p25 and p35 expression may result in a loss of inhibition of GSK-3 which should lead to tau hyperphosphorylation. In agreement with this idea, inhibitory crosstalk between Cdk5 and GSK-3 was demonstrated (Plattner et al., 2006; Wen et al., 2008). Thus, reduced p25 expression is expected to lead to an overactivation of GSK-3 activity that causes tau hyperphosphorylation. This scenario would be consistent with other evidences that GSK-3 may be a critical tau kinase in AD (Hooper et al., 2008).

## CONCLUDING REMARKS

In recent years it has emerged that p25 is not an exclusively toxic molecule. Spatial memory formation leads to p25 generation in the hippocampus, where p25 can stimulate synthesis of synaptic proteins followed by synaptogenesis and synaptic strengthening. In the early stages of AD hippocampal p25 expression is reduced. Taken together, this suggests that p25 generation is a memory mechanism that is affected in early AD (**Figure 1**). Impaired p25 generation in AD is expected to contribute to synaptic degeneration in the disease. However, more studies are needed to establish the precise synaptic function of p25. For example, p25 generation could be a signal from synapse to nucleus to stimulate gene expression needed for long-lasting synaptic plasticity. Recent evidence also shows that the expression of p25-regulated synaptic proteins is downregulated in early AD hippocampus. Analysis of these synaptic downregulations promises to give insight into the mechanisms of early synaptic degeneration in AD. So far, it has emerged that p25-regulated proteins are downregulated at the pre- and post-synaptic side, suggesting that degeneration occurs equally at both sides of the synapse. This is consistent with the finding that oligomeric amyloid peptide binds to pre- and post-synapses and has the potential to induce dysfunction on both sides of the synaptic cleft (Koffie et al., 2012). It is hoped that in future a mechanistic understanding of synaptic degeneration in AD will lead to the development of pharmacological approaches to contain this devastating disease in its early stages.

## Conflict of Interest Statement

The author declares that the research was conducted in the absence of any commercial or financial relationships that could be construed as a potential conflict of interest.
